# Computational fluid dynamics models and congenital heart diseases

**DOI:** 10.3389/fped.2013.00004

**Published:** 2013-02-26

**Authors:** Giancarlo Pennati, Chiara Corsini, Tain-Yen Hsia, Francesco Migliavacca, for the Modeling of Congenital Hearts Alliance (MOCHA) Investigators

**Affiliations:** ^1^Laboratory of Biological Structure Mechanics, Chemistry, Materials and Chemical Engineering Department “Giulio Natta”, Politecnico di MilanoMilano, Italy; ^2^Cardiac Unit, Great Ormond Street Hospital for ChildrenLondon, UK; MOCHA Investigators: Edward Bove MD and Adam Dorfman MD (University of Michigan, USA); Andrew Taylor MD, Alessandro Giardini MD, Sachin Khambadkone MD, Jam Marek MD, Silvia Schievano PhD, and T-Y Hsia MD (Institute of Child Health, UK); G. Hamilton Baker MD and Anthony Hlavacek (Medical University of South Carolina, USA); Francesco Migliavacca PhD, Giancarlo Pennati PhD, and Gabriele Dubini PhD (Politecnico di Milano, Italy); Richard Figliola PhD and John McGregor PhD (Clemson University, USA); Alison Marsden PhD (University of California, San Diego, USA); Jeff Feinstein MD (Stanford University, USA); Irene Vignon-Clementel (National Institute of Research in Informatics and Automation, France)

**Keywords:** mathematical model, fluid dynamics, patient-specific, medical images

## Abstract

Mathematical modeling is a powerful tool to investigate hemodynamics of the circulatory system. With improving imaging techniques and detailed clinical investigations, it is now possible to construct patient-specific models of reconstructive surgeries for the treatment of congenital heart diseases. These models can help clinicians to better understand the hemodynamic behavior of different surgical options for a treated patient. This review outlines recent advances in mathematical modeling in congenital heart diseases, the discoveries and limitations these models present, and future directions that are on the horizon.

## Introduction

Treatment of congenital heart diseases (CHD) has evolved rapidly in recent years, requiring customized therapies due to the large inter-patient variability of anatomical and hemodynamic parameters within the vascular arrangement following a surgical repair. Virtual surgery based on computational fluid dynamics or *in silico* patient-specific modeling is a promising tool because it could help surgeons in the decision-making process, improving and understanding hemodynamic outcomes and reducing trial errors during complex surgeries (1–4).

The major areas of research for mathematical modeling of CHD are univentricular circulation and aortic and pulmonary malformations. The former is the focus of this article, while a detailed review on aortic coarctation has been provided recently by LaDisa et al. (5).

## Univentricular circulation modeling

Palliations developed to treat single ventricle heart defects aim to by-pass the non-functional ventricle and establish a direct connection between the systemic venous and pulmonary arterial circulations (6). These treatments usually include three consecutive stages: (i) Norwood procedure [Blalock–Taussig shunts, central shunts, right ventricle-to-pulmonary artery conduit and the hybrid Norwood (7)], (ii) Glenn or hemi-Fontan procedures, and (iii) the complete Fontan procedure (or total cavo-pulmonary connection). The peculiar geometries resulting from these surgical procedures are associated with different fluid dynamics that may affect the univentricular circulation with different energy losses and uneven blood distribution to the lungs.

**Figure 1:**
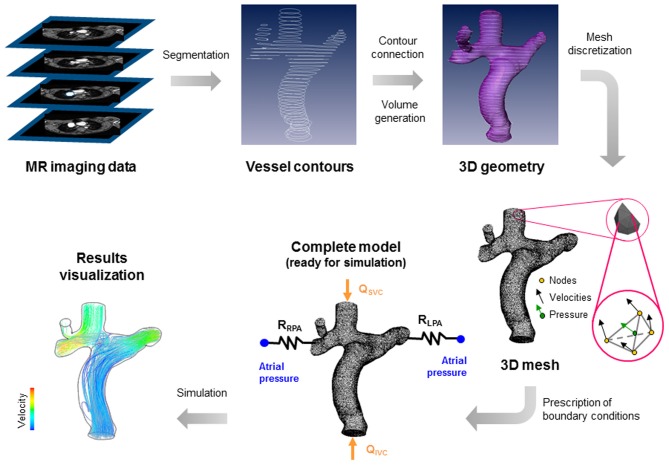
**Steps required to build a patient-specific model of cavo-pulmonary connection.** (1) Acquisition of clinical images (from magnetic resonance); (2) extraction of vessel contours; (3) generation of 3D vessel surface and volume; (4) discretization of the geometry in small elements or volumes; (5) prescription of boundary conditions (in this case the superior and inferior venae cavae flows—Q_SVC_ and Q_IVC_—and atrial pressure); (6) simulation and visualization of results (in this case particle paths, colored by velocity, injected from superior and inferior venae cavae).

The up-to-date modeling approach to study these surgeries is based on different steps summarized in Figure 1, where a total cavo-pulmonary connection is shown as an example. First, the clinical images, generally from magnetic resonance (MR) or computed tomography (CT), are piled-up and used to extract the vessel contours. Secondly the generated curves are connected to create a three-dimensional (3D) geometry that can be eventually smoothed. The resulting geometry is then divided in small elements or volumes. This process is called mesh discretization. In the vertexes or centers of the elements or volumes, the governing fluid dynamics equations (i.e., Navier-Stokes equations) are solved, imposing proper boundary conditions and allowing the visualization of velocities, pressures fields, and the quantities that can be derived from them, e.g., the wall shear stresses. For a more detailed description on how these models work and can be constructed we refer the reader to the works by Vignon-Clementel et al. (8) and Corsini et al. (4).

From a modeling point of view the study of fluid dynamics in systemic-to-pulmonary shunts is a more complex and challenging issue than that related to the later surgical stages, where the flows are less pulsatile and pressure fields are more uniform. In fact, fluid dynamics in stage one has rarely been investigated and modeling works represent a minority among all those related to surgical repairs for treating CHD. One reason is that the shunt and surrounding geometry after the operation varies a lot among individuals, and the shunt flow, typically the only source of pulmonary flow, is pulsatile with significant pressure gradients and velocities. An accurate mathematical description or numerical simulation of the local fluid dynamics of the shunt is thus necessary in modeling the Norwood circulation as the shunt flow is driven by the pressure difference between the systemic and pulmonary arterial circulations, and is regulated by the geometrical shunt features. Nevertheless, any hemodynamic analysis cannot be localized on the shunt region alone, since the whole circulation has to be accounted for in order to ensure proper coronary perfusion, minimize ventricular volume overload, and pulmonary hypertension. Furthermore, in infants and small babies, mathematically accurate measurements and data needed for modeling purposes are difficult to obtain. Most studies have been limited to either 3D computational models (9–14) or *in vitro* models (15–19) of the local fluid dynamics. Such models, although providing a very detailed description of the local hemodynamics, could not describe the mutual interactions with the remainder of the circulatory system, because boundary conditions (flows or pressures) were enforced to the model.

A first attempt to remove this limitation was made by Migliavacca and colleagues (20), who built a detailed closed-loop lumped parameter, i.e., zero-dimensional, model (LPM or 0D) accounting for the entire circulation of the patient. In particular, the complex hydraulic behavior of the shunt was modeled as a non-linear resistance varying with the shunt diameter and flow rate, whose parameter values had been previously deduced from simulations with 3D local models. Although simulation results matched clinical observations reasonably well, the lack of clinical data (except for the whole systemic and pulmonary vascular resistances) measured in peripheral regions of the circulation made parameter identification a difficult task.

Later on, in order to utilize the information obtained from both 3D models (i.e., local fluid dynamic variables and effect of changing anatomical features) and LPMs (i.e., pressures and flow distribution throughout the peripheral circulation) at the same time, a combined approach coupling the two types of models has been developed. A computational application of this methodology can be found in Laganà et al. (21), Migliavacca et al. (22), Bove et al. (23), Baretta et al. (24) and Corsini et al. (25), where the hemodynamic effects of a central shunt, a modified Blalock–Taussig shunt, a right ventricle-to-pulmonary artery shunt and the hybrid approach were analyzed in terms of pulmonary and coronary perfusion, as well as ventricular performance.

Despite the advantages brought by the combined methodology into the study of surgical procedures whose hemodynamic outcomes are still hard to predict from a clinical point of view, and given that a complete customization of the cardiovascular system is not achievable, modeling of shunts so far is limited to designs, whether 3D or 0D, which refer to clinical data averaged over groups of patients. This could be partly solved using MR scans and hemodynamic data from Doppler, MR or catheterization exams performed on a specific patient in preparation for the next surgical stage, i.e., Glenn procedure (26).

Contrary to the Norwood circulation, a number of *in vitro* and *in silico* studies evaluated the local and global fluid dynamics following the surgical creation of cavo-pulmonary anastomoses, in order to find the design that minimizes energy dissipations and optimizes the flow balance between the right and left pulmonary arteries (RPA and LPA). A review of the literature related to this topic is reported in DeGroff (27). Earlier studies reconstructed simplified geometries, representing typical anatomies of patients with cavo-pulmonary connections (28–33). More recently, the use of tomographic medical imaging techniques, such as MR and CT, has joined the well-established ultrasound and angiography imaging techniques and has allowed more realistic 3D reconstructions of a specific patient's vessels (34–39).

However, the sole geometrical accuracy does not guarantee realistic haemodynamic results. Indeed, boundary conditions required by *in silico* models may play a major role in determining energy dissipations and controlling flow distribution among the pulmonary branches (33, 37, 40–42).

In several *in silico* and *in vitro* studies, either volume flow rates or velocity profiles were prescribed as inlet boundary conditions together with a fixed pressure at the pulmonary outlets. As an alternative, flows were employed to set proper outlet boundary conditions (i.e., flow ratio to lungs) as well. This allows the user to define, apply or calculate different lung resistances of the model, not necessarily assuming that the right and left pulmonary vascular resistances (PVRs) are identical. In most of the recent published works, inlet and outlet flow values were assumed on the basis of averaged or time dependent clinical measurements. For example, use of MR phase contrast velocity mapping provides volume flow rates for the vessels supplying (SVC and, in case, IVC) and draining (LPA and RPA) the region of the cavo-pulmonary connection, thus allowing the simulation of patient-specific hemodynamics and power loss calculation (36, 43, 44).

In patient-specific modeling of cavo-pulmonary connections, the prescription of realistic outlet boundary conditions is necessary since they determine blood flow split into the RPA and LPA. PVR can be clinically evaluated through a catheterization exam where atrial and pulmonary arterial pressures are measured, but measurements of the individual pulmonary arterial branches (RPA and LPA) are usually absent due to the poor resolution of pressure sensors and the uncertainty of catheter tip location. Therefore, if left and right pulmonary blood flows are not available from MR data, a common assumption is to adopt equal values for left and right PVRs (29, 45, 46).

More appropriate patient-specific impedance values can be deduced on the basis of numerical simulations taking into account both pressure and flow collected data. Marsden et al. (37) used a coupled multidomain method (47) to apply resistance boundary conditions at the pulmonary outlets of 3D models of cavo-pulmonary connections reconstructed by means of MR and CT images. However, a number of important assumptions were done due to lack of clinical measurements: (i) left-right lung flow split was assumed; (ii) pulmonary resistances were chosen to obtain a pressure level in the SVC in agreement with cardiac catheterization data; (iii) left and right resistance values were assumed to be either inversely proportional to the outlet areas of the model or different fractions of the left/right pulmonary flows imposed to the pulmonary lobes.

An interesting approach for tackling the problem of outlet boundary conditions in pulmonary stenotic models was proposed by Spilker and colleagues (40) who coupled a patient-specific 3D finite element model of the proximal pulmonary arteries, with a one-dimensional linearized morphometric representation of the distal lung vasculature with diameters down to 20 μm, i.e., slightly larger than capillaries. Recently, Spilker and Taylor (42) have developed an automatic and effective method to systematically tune the LPMs used as outlet boundary conditions of 3D blood flow models (i.e., an idealized common carotid artery, an idealized iliac arterial bifurcation, and a patient-specific abdominal aorta) in order to achieve the desired features of pressure and flow waveforms. Hence, the most recent strategy suggested the use of patient-specific values not only to impose the inlet flow, but also to derive the downstream impedances coupled to patient-specific 3D anatomic models.

However, if future scenarios such as after a surgical geometry modification (35, 48) or exercise conditions (37) are simulated, actual flow rates are not available. Although usually adopted, pre-operative blood flows might be very different from those occurring in new scenarios, thus preventing an accurate virtual planning of the cardiovascular surgical repairs. Once again, combined modeling approaches which couple a 3D model of the surgical region with a LPM (22, 42, 44, 49–52) or a one-dimensional model (40) of the remainder of the cardiovascular system may solve the issue of defining reasonable boundary conditions, provided that a closed-loop coupling is performed.

The predictive role of the virtual surgery in helping surgeons choose the most effective customized procedure might be useful for the treatment of CHD, though it implies several challenges (44), and requires a considerable, interactive work among surgeons, physicians and engineers. The main limitations affecting computational modeling of CHD are: the MR imaging resolution, the wall rigidity assumption, and the absence of implementation in the LPM of short-term feed-back mechanisms (e.g., the influence of coronary perfusion on cardiac contractility) and long-term adaptive phenomena (e.g., physiologic/pathologic changes due to vessel remodeling and patient growth). Even with the state-of-the-art imaging techniques, the first limitation still impedes an accurate 3D modeling of small vessels or conduits such as the systemic-to-pulmonary shunts. The second point should be considered especially for the first stage palliation, since previous experimental (18) and computational (11) studies suggested that the shape and area of the distal anastomosis of the shunt significantly changes with the pulmonary artery pressure, thus with the PVR. Therefore, shunts with different diameters do not necessarily behave in different ways, depending on their downstream resistance. However, simulating compliant vessels with complex geometry and steeply changing wall properties (from systemic artery to Gore-Tex, to pulmonary artery) is not trivial and would require a noticeable computational effort. To overcome the last limitation implies deep knowledge of the patho-physiologic mechanisms causing the aforementioned phenomena, but enough relevant clinical data allowing realistic modeling are still not available.

Finally, a brief note should be devoted to the fluid dynamics generated by mechanical devices. In this context, blood pump specifically designed to support adolescent and adult patients with single ventricle physiology has been recently studied with the aim of providing a therapeutic option to medically stabilize patients. Rodefeld et al. (53) presented a 3D idealized computational model of the total cavo-pulmonary connection with an impeller represented as a smooth two-sided conical actuator disk with rotation in the vena caval axis. The authors demonstrated a reduction in power loss by 88%. More recently the same authors (54) investigated the hemodynamic and hemolysis performance of a catheter-based viscous impeller pump to power the Fontan circulation either with computer simulations and a mock circuit.

## Aortic and pulmonary malformations

Mathematical modeling has also been applied with success to the studies of aortic and pulmonary malformations.

**Figure 2:**
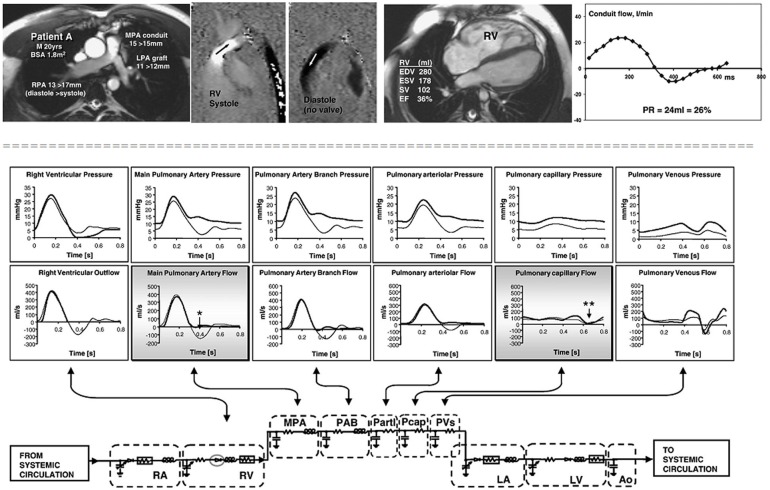
**Cardiovascular magnetic resonance studies in a patient without an effective pulmonary valve and pulmonary regurgitation.** The patient (upper panels) was a 20 year old man, born with tetralogy of Fallot, palliated with a right Blalock–Taussig shunt and repaired at 8 years with a homograft right ventricle to pulmonary artery conduit and graft augmentation of the proximal left pulmonary artery. Cine imaging (upper row, first panel) and inplane, vertically encoded velocity maps aligned with the right ventricular outflow tract (upper row, second and third panel). No effective valve action in diastole. Flow curve (upper row, fourth panel) plotted from retrospectively gated acquisitions of velocities through planes transecting the proximal MPA, giving the regurgitant volumes and fractions shown. The peak systolic velocity was 3 m/s in the conduit. Fifth panel reports the right ventricular volume measurements. Lower panel: results from a lumped parameter model describing the pulmonary circulation. The right heart and pulmonary vascular components of the numerical model represented by electrical analog symbols with plots of pressure and flow through each part of the model calculated with and without pulmonary valve function (broad and thin lines, respectively). Without the valve, there is regurgitant flow in early diastole at main pulmonary artery level (^*^) with late diastolic forward flow, but there is no reversal of flow at capillary level (^**^), which means that the regurgitant volume originates entirely from compliance of the virtual pulmonary arteries and arterioles. M, male; BSA, body surface area; MPA, main pulmonary artery; RPA, right pulmonary artery; LPA, left pulmonary artery; RV, right ventricle; PR, pulmonary regurgitation; EDV, end diastolic volume; ESV, end systolic volume; SV, stroke volume; EF, ejection fraction. RA, right atrium; RV, right ventricle; PAB, pulmonary artery branches; Partl, pulmonary arterioles; Pcap, pulmonary capillaries; PVs, pulmonary veins; LA, left atrium; LV, left ventricle; Ao, aorta. (With permission from 55).

Stand-alone lumped parameters models have demonstrated a good performance in the study of pulmonary regurgitation after repair of tetralogy of Fallot (55). The authors demonstrated that amount of regurgitation, in the absence of an effective valve, depends on pulmonary arterial compliance and on the location of resistance relative to the compliance (Figure 2).

LaDisa et al. (5) reviewed the recent modeling literature on aortic coarctation similarly to what applied in the univentricular circulation. The results from the analysed studies provided a greater understanding of the preoperative and postoperative assessment of surgical and interventional options and an additional tool for prediction of postoperative quantities. Aortic walls are compliant and the interaction between the fluid and solid parts is more important than in the venous districts. For these reasons the most recent studies (56) included also a fluid-structure interaction (FSI) modeling.

## Conclusions

In mathematical modeling of surgical corrections for univentricular hearts, pulmonary and aortic malformations, several goals have been achieved so far, namely:

- Availability of patient-specific imaging data to allow the reconstruction of customized 3D models;
- flow, velocity and pressure data acquired from the investigated patient (with MR, Doppler and catheterization exams) are used to define patient-specific boundary conditions;
- connecting a LPM to a geometrical model in a closed-loop fashion enables mutual interaction between the local (more detailed) and global descriptions of the investigated fluid dynamics, thus avoiding unrealistic assumptions on the upstream and downstream circulations;
- the modeling software currently available allows to perform various virtual surgeries on the studied anatomic models, simulating the final result of the surgical operation; such models can be used further to evaluate which is the best model in terms of hemodynamic performance;
- with the current computing power availability in computational laboratories, simulations tasks that used to require days to complete can now be solved in hours.

In the future, however, some aspects of the modeling process need to be refined:

- Both imaging (e.g., from MR) resolution and geometrical refining algorithms currently used are expected to improve, in order to reconstruct more faithfully customized complex anatomies and tiny vessels;
- virtual surgical planning needs to emphasize the importance of pre-operatively collecting as many congruent patient-specific data as possible, to predict more accurately the patient-specific hemodynamics in the new configurations;
- relevant clinical data should be collected also to explore the causes of short-term and long-term adaptive phenomena, with the final objective of their realistic implementation within the models;
- for application in a clinical environment computing power should further increase to allow simulation times to be in the order of minutes.

Information obtained from numerical models cannot substitute clinical exams and judgment, but can provide insights to guide clinical decisions. Furthermore, allowing predictions and interpretations of phenomena and mechanisms that are not well-defined, mathematical models can resolve complex, altered cardiovascular physiology, as well as evaluate clinically relevant parameters which are not routinely measured. The added value resulting from computational modeling has already been exploited in the clinical management of coronary artery disease in adults (57). This could be potentially applied to CHD, provided that proper accuracy and resolution are obtained with advancements in the measurement and imaging techniques.

### Conflict of interest statement

The authors declare that the research was conducted in the absence of any commercial or financial relationships that could be construed as a potential conflict of interest.
